# Immunotherapy of Neuroendocrine Neoplasms: Any Role for the Chimeric Antigen Receptor T Cells?

**DOI:** 10.3390/cancers14163991

**Published:** 2022-08-18

**Authors:** Giuseppe Fanciulli, Roberta Modica, Anna La Salvia, Federica Campolo, Tullio Florio, Nevena Mikovic, Alice Plebani, Valentina Di Vito, Annamaria Colao, Antongiulio Faggiano

**Affiliations:** 1 Neuroendocrine Tumour Unit, Department of Medicine, Surgery and Pharmacy, University of Sassari—Endocrine Unit, AOU Sassari, 07100 Sassari, Italy; 2 Endocrinology, Diabetology and Andrology Unit, Department of Clinical Medicine and Surgery, Federico II University of Naples, 80131 Naples, Italy; 3 Division of Medical Oncology 2, IRCCS Regina Elena National Cancer Institute, 00144 Rome, Italy; 4 Department of Experimental Medicine, “Sapienza” University of Rome, 00161 Rome, Italy; 5 Department of Internal Medicine, University of Genoa, 16132 Genoa, Italy; 6 Scientific Institute for Research, Hospitalisation and Healthcare Ospedale Policlinico San Martino, 16132 Genoa, Italy; 7 Endocrinology Unit, Department of Clinical and Molecular Medicine, Sant’Andrea Hospital, ENETS Center of Excellence, Sapienza University of Rome, 00189 Rome, Italy; 8 Laboratory of Geriatric and Oncologic Neuroendocrinology Research, Istituto Auxologico Italiano IRCCS, Cusano Milanino, 20095 Milan, Italy; 9 UNESCO Chair, Education for Health and Sustainable Development, Federico II University, 80131 Naples, Italy

**Keywords:** chimeric antigen receptor-T cells, neuroendocrine neoplasm, neuroendocrine tumor, neuroendocrine carcinoma, carcinoid tumor, somatostatin receptors

## Abstract

**Simple Summary:**

Neuroendocrine neoplasms (NENs) comprise a heterogeneous group of tumors arising in different organs whose clinical course is variable according to histological differentiation and metastatic spread. Therapeutic options have recently expanded, but there is a need for new effective therapies, especially in less differentiated forms. Chimeric antigen receptor T cells (CAR-T) have shown efficacy in several cancers, mainly hematological, but data on NENs are scattered. We aimed to analyze the available preclinical and clinical data about CAR-T in NENs, to highlight their potential role in clinical practice. A significant therapeutic effect of CAR-T cells in NENs emerges from preclinical studies. Results from clinical trials are expected in order to define their effective role in these cancers.

**Abstract:**

Neuroendocrine neoplasms (NENs) are a heterogeneous group of tumors with variable clinical presentation and prognosis. Surgery, when feasible, is the most effective and often curative treatment. However, NENs are frequently locally advanced or already metastatic at diagnosis. Consequently, additional local or systemic therapeutic approaches are required. Immunotherapy, based on chimeric antigen receptor T cells (CAR-T), is showing impressive results in several cancer treatments. The aim of this narrative review is to analyze the available data about the use of CAR-T in NENs, including studies in both preclinical and clinical settings. We performed an extensive search for relevant data sources, comprising full-published articles, abstracts from international meetings, and worldwide registered clinical trials. Preclinical studies performed on both cell lines and animal models indicate a significant therapeutic effect of CAR-T cells in NENs. Ongoing and future clinical trials will clarify the possible role of these drugs in patients with highly aggressive NENs.

## 1. Introduction

**Figure 1:**
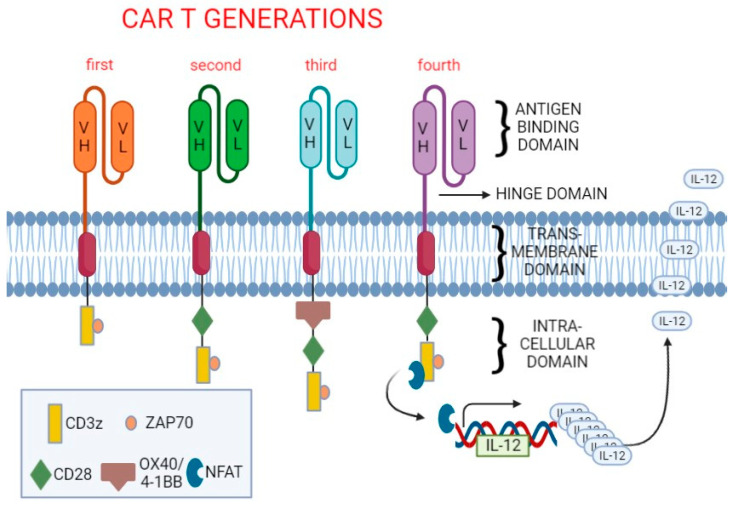
Diagrammatic representation of chimeric antigen receptors (CARs).

Neuroendocrine neoplasms (NENs) include a heterogeneous group of tumors with variable clinical presentation and prognosis, mainly arising in the gastroenteropancreatic and pulmonary tract, with steadily increasing incidence, irrespective of site and stage [1,2]. Even though NENs usually show an indolent behavior, they are often locally advanced or already metastatic at diagnosis [1,2]. Their management is therefore challenging, and, even though the landscape of therapy has considerably expanded in the last decades [3,4], additional loco-regional or systemic approaches are required [5]. The available treatment options include somatostatin analogues (SSAs), such as octreotide and lanreotide, targeted therapies (everolimus and sunitinib), liver-directed therapies, external beam radiotherapy, peptide receptor radionuclide therapy, and chemotherapies, in variable sequences [6,7]. Prognosis is largely influenced by several factors, including patient age, tumor grade, stage, and localization [2]. Although survival rates have been improving over time, tailored therapies are needed, especially for patients with rapidly progressing diseases. The role of immunotherapy in NENs is gaining interest [8,9], and encouraging clinical results have been reported in small cell lung cancer (SCLC) [10], Merkel cell carcinoma [11], pheochromocytoma/paraganglioma [12], lung carcinoid [13], and medullary thyroid carcinoma (MTC) [14]. T cell immunotherapy using CAR-T cells is showing encouraging results in cancer treatment. CARs are recombinant receptors composed of the single-chain fragment variant (scFv) of an antibody for recognition of specific antigens, an extracellular hinge domain, a transmembrane domain, and an intracellular signal domain (Figure 1) [15]. A CAR-T cell is a T lymphocyte isolated from the patient, genetically engineered to express an antigen-specific receptor, introduced using a plasmid or viral vector, that binds directly to a cell surface antigen expressed on the cell intended to be recognized and eliminated [16]. The T cells used for CAR-T cell engineering are obtained from patient’s peripheral blood cells, which are expanded ex vivo and then re-infused back in patients after lymphodepleting chemotherapy. After binding to target antigens, CAR-T cells are activated, proliferate, and exert their antitumor activity, which includes tumor lysis and induction of a secondary immune response against the tumor [17]. CAR-T cells have demonstrated remarkable success in the treatment of hematological tumors, and, to date, the European Medicines Agency (EMA) and US Food and Drug Administration (FDA) have approved six different CAR-T cell immunotherapies for these malignancies (Table 1).

CARs are transduced on the T cell surface and interact with the selected antigens on tumor cells to induce T cell-mediated cytotoxicity. CARs are composed of a single-chain variable fragment (antigen-binding domain) composed of the variable domains, VH and VL, of the heavy and light chains of a monoclonal antibody connected via a long flexible linker to form a single-stranded fragment, which is able to recognize specific proteins on tumor cells. This portion is connected to a transmembrane domain via a hinge domain, structurally reproducing sequences of CD8α, CD28, or the Fc region of the immunoglobulins IgG1 or IgG4. The intracellular signaling domains represent the anchor of the molecule to the cell membrane and the connections with the intracellular signaling domain. This latter domain is composed of the element initiator of the T cell response, CD3-ζ, and it has been implemented over the years to enhance the cytotoxic response with the development of four different generation of CARs. While the first generation included the CD3-ζ domain only, in the subsequent ones, a costimulatory signal from CD28 (second generation) or multiple costimulatory elements besides CD28, including also 4-1BB or OX40, were introduced (third generation). Third-generation CAR-T cells showed increased differentiation towards T cell effectors, prolonged T-cell survival, and better clinical outcomes. A further development (fourth generation) of CARs was obtained by adding to the costimulatory signals the presence of transcription factors, such as NFAT, able to activate innate immune response through the production and secretion of IL-12 or other pro-inflammatory cytokines. The improved efficacy of third vs. first generation CDH17-targeting CAR-T cells was also observed in preclinical in vitro and in vivo studies against gastrointestinal NET cells (see text for detailed description).

The success and the continuous advancements achieved with CAR-T cells in hematological malignancies have encouraged their use in solid tumors, and indeed significant responses in patients with solid malignancies have been reported [18]. On this basis, we aimed to collect and discuss the available data about the CAR-T cell treatment in NENs, including both preclinical and clinical settings.

## 2. Search Strategy

We performed an extensive search for relevant data sources, including full-published articles in international online databases (PubMed, Web of Science, Scopus) and preliminary reports in selected international meeting abstract repositories (American Society of Clinical Oncology, ASCO; European Neuroendocrine Tumor Society, ENETS; European Society for Medical Oncology, ESMO), or short articles published as supplements of international meetings, by using the following terms: chimeric antigen receptor-T cells, CAR-T cells, receptors chimeric antigen, neuroendocrine neoplasm, neuroendocrine tumor, neuroendocrine carcinoma, carcinoid tumor, small cell lung cancer, large cell lung cancer, lung carcinoid, gastroenteropancreatic neuroendocrine tumor, gastroenteropancreatic neuroendocrine neoplasm, pheochromocytoma, paraganglioma, medullary thyroid carcinoma, pituitary tumor, somatostatin receptors. By using the same keywords adopted for reviewing published articles, we conducted an in-depth search on Registered Clinical Trials (RCTs) by using the US National Institutes of Health registry of clinical trials (http://clinicaltrials.gov) and any primary register of the World Health Organization (WHO) International Clinical Trials Registry Platform (ICTRP), http://www.who.int/ictrp/network/en/. The search was last updated on 30 June 2022.

The search revealed data on MTC, SCLC, Neuroendocrine prostate cancer (NEPC), pancreatic NENs (pNENs), and ileal and lung neuroendocrine cells.

### 2.1. Medullary Thyroid Carcinoma

MTC is a neuroendocrine tumor arising from calcitonin-producing parafollicular C cells of the thyroid gland, accounting for approximately 3–5% of all thyroid malignancies [19,20]. Most cases of MTC are sporadic; however, up to 25% are associated with a hereditary mutation in the REarranged during Transfection (RET) proto-oncogene [20]. Although rare, the incidence of MTC has significantly increased over the last 3 decades from 0.14 to 0.21 per 100,000 individuals, accounting for 13.4% of the total deaths attributable to thyroid cancer [21]. MTC presents with loco-regional metastasis in up to 50% of patients, distant metastasis in 10–15%, and recurrent disease develops in approximately 50% of patients [22]. The clinical course of patients with MTC is variable, ranging from mild to extremely aggressive, with a 5-year survival rate of less than 40% [23]. According to the American Thyroid Association guidelines, total thyroidectomy and dissection of cervical lymph node compartments represent the standard treatment for sporadic or hereditary MTC, but the management of advanced and progressive disease remains challenging [24]. Systemic chemotherapy has shown limited efficacy in metastatic MTC, and SSAs and everolimus have been proposed with promising results [25]. Two oral tyrosine kinase inhibitors (TKI), vandetanib and cabozantinib [26], have been approved for progressive or symptomatic MTC, showing improvement in progression-free survival (PFS) but no increase in overall survival (OV). Notably, these drugs are nonselective, and toxicity due to off-target effects is not negligible [27]. The National Comprehensive Cancer Network (NCCN) guidelines recommend that TKI therapy may not be appropriate for stable or slowly progressive, indolent disease [28]. More recently, the highly selective RET inhibitors selpercatinib (LOXO-292) and pralsetinib (BLU-667), showed efficacy in advanced MTC, but molecular testing for germline or somatic RET mutations is essential [29]. Thus, a strong need for advanced MTC effective therapies still remains. Immunotherapy represents another approach that might be better explored in the treatment of MTC. In this context, the use of chimeric antigen receptor technology could open a promising therapeutic scenario. Based on the expression of GFRα4 by MTC, Bhoj and colleagues [27] hypothesized that GFRα4 might be a putative target antigen for CAR-based T cell immunotherapy for MTC. Using phage display, they constructed P4-10bbz, a CAR that specifically targets GFRα4. This construct was cloned into a lentiviral plasmid vector, and through viral transduction GFRα4 targeting CAR-T cells were engineered. In vitro experiments showed that P4-10bbz CAR specifically responded to GFRα4. P4-10bbz CAR was expressed in a Jurkat cell line expressing an NFAT (nuclear factor of activated T-cells)-driven Green Fluorescent Protein (GFP) reporter construct. Measuring GFP expression by flow cytometry, P4-10bbz expressing Jurkat cells showed activation over basal levels when grown in wells coated with recombinant or containing soluble GFRα4 as well as when co-cultured with TT and MZ-CRC1 cells (two human MTC cell lines, which express the target antigen GFRα4). P4-10bbz CAR was also expressed in primary human T cells. When these P4-10bbz CAR-T cells were co-cultured with TT and MZ-CRC1 cells, they caused lysis of 60–70% of the cells. Increasing levels of expressed interleukin-2 (IL-2) and interferon γ (IFNγ) were detected by ELISA in the conditioned media when P4-10bbz CAR-T cells are co-cultured with GFRα4 expressing cells, further highlighting an activation of CAR-T cells. Bhoj and colleagues also demonstrated the in vivo efficacy of P4-10bbz CAR-T cells against MTC, using an MTC xenograft mouse model. The tumor mass volume, assessed by bioluminescence imaging, significantly decreased after CAR-T cell treatment. At the same time point, peripheral blood human T cells were counted by flow cytometry, showing that antitumor activity was accompanied by robust T cell expansion. In conclusion, P4-10bbz CAR-T cells effectively eradicated antigen-positive tumor cells. The response to GFRα4 is now under study in humans: NCT04877613 is an open-label phase 1 study aimed to assess the safety of different doses of GFRα4 CAR-T cells in adult patients with recurrent/metastatic MTC (progressive after at least one prior TKI-containing regimen, or in patients that were intolerant to or declined such therapy). Fludarabine and cyclophosphamide were used to induce lymphodepletion. The primary outcome is the incidence of treatment-emergent adverse events, as assessed by CTCAE v5.0. Among the secondary outcomes measures, Duration of Response, OS, and PFS are included. The study start date was 19 August 2021, with an estimated enrolment of 18 participants, and it is presently reported as “recruiting”. Study completion is expected on 1 June 2039.

### 2.2. Small Cell Lung Carcinoma

SCLC is an aggressive, poorly differentiated, and high-grade neuroendocrine carcinoma (HG-NEC) that accounts for 15% of all lung carcinomas [30]. The incidence of SCLC has decreased in recent decades, with a prevalence of 1–5 per 10,000 people in the European community. According to the 2021 WHO classification of thoracic tumors [31], SCLC and large cell neuroendocrine carcinomas are the two lung HG-NEC subtypes sub-classified on the basis of cellular size. SCLC is the most frequent neuroendocrine lung cancer, which is commonly diagnosed as an advanced-stage disease. The gold standard treatment strategy for early-stage SCLC patients is the complete resection with mediastinal lymph node dissection [32]. However, unfortunately, the disease often rapidly recurs, with a reported rate exceeding 50%. In the setting of chemonaïve locally advanced/metastatic disease, the standard of care has been radically modified in the last decade [33]. Anti-programmed cell death-1 (anti-PD-1)/anti-programmed cell death ligand-1 (anti-PD-L1) immune checkpoint inhibitors have been incorporated into treatment algorithms for advanced SCLC [34]. This change has been made on the basis of several randomized clinical trials, demonstrating a consistent OS benefit with the early addition of immunotherapy (atezolizumab, IMPOWER 133; durvalumab, CASPIAN; pembrolizumab, Keynote-604) to platinum-based chemotherapy (cisplatin or carboplatin plus etoposide) plus chemotherapy for SCLC patients [35,36,37]. However, unfortunately, the overall prognosis of SCLC patients remains unfavorable, with a median OS of 10–12 months and one- and two-year OS rates of 56.2% and 21.7%, respectively [38]. Therefore, the search for more personalized, innovative, and effective therapies represents an unmet need in this context. In 2021, Taromi and colleagues analyzed the role of AC133-specific CAR-T cells for SCLC in vitro and in vivo preclinical models [39]. The AC133 epitope of CD133 has been identified as a potential target for CAR-T cells, given that the relapse of SCLC has been demonstrated to be caused by cancer stem cells (CSC) that express glycosylated AC133 form [40]. In this study, the authors carried out an assessment of the AC133-specific CAR-T cells in an orthotopic SCLC murine model as well as in SCLC cell lines. First, the authors yielded the generation of CAR-T cells using the AC133scFv derived from the anti-SC133.1mAB. Then, through cytotoxicity assays, the authors demonstrated that the CAR-T cells specifically lysed AC133-positive SCLC cells. Additionally, they demonstrated that AC133-specific CAR-T cells were able to infiltrate SCLC xenografts in vivo. Interestingly, the rates of infiltration were higher after the administration of chemotherapy to the murine models. Moreover, AC133-specific CAR-T cells determined a reduction of the tumor burden measured by magnetic resonance imaging in mice. The xenografted mice treated with AC133-specific CAR-T cells also presented a longer survival if compared to the ones who did not receive the experimental treatment. In addition, the combination of AC133-specific CAR-T cells and anti-PD-1 therapy was tested, demonstrating a synergic activity. Finally, the authors combined the AC133-specific CAR-T cells with anti-PD-1 and a third compound, a CD73 inhibitor. By using these three agents together, a further improvement in survival rates was achieved in this in vivo model of SCLC, with long-term control of the tumors. A recent study carried out by Reppel et al., evaluated the effect of disialoganglioside GD2 (GD2) CAR-T both in SCLC cells lines as well as in vivo xenograft models of primary and metastatic tumors from SCLC [41]. In fact, a hyperexpression of GD2 has been detected in SCLC cells, and it has been identified as a potentially relevant therapeutic target for immunotherapy in lung neuroendocrine cancers. GD2-CAR-T cells were obtained incorporating interleukin 15 (IL-15) in order to support CAR-T cell expansion and persistence over time. The structure of the GD2-CAR was generated using the scFv derived from the 14G2a mAb. In addition, a cassette was generated, encoding either the optimized GD2. CAR in combination with IL-15 (GD2.CAR.15) using a 2A-sequence peptide [42]. In vitro experiments demonstrated that GD2 CAR-T cells eliminated GD2 positive cells. GD2 CAR-T cells were also shown to target GD2 + SCLC in orthotopic xenograft models. Finally, through the addition of the EZH2 inhibitor tazemetostat, the authors obtained an upregulation of GD2 and an improved susceptibility to the cytotoxic effects of GD2-specific CAR-T cells. Another CAR-T cell construct developed by Crossland et al. in 2018 [43] targeted CD56, also named NCAM-1 (neuronal cell adhesion molecule 1), a glycoprotein highly expressed on the surface of malignancies with a neuronal or neuroendocrine origin, including SCLC, independently of HLA expression. CD56-CART showed significant cytolytic activity against SCLC CD56+ cell lines in vivo and in vivo. This molecule has already been the target of different antibody-based therapeutic strategies and has been proven to show antitumoral activity in previous preclinical models for different malignancies [44]. Through their work, the authors showed that CD56 CAR-T cells lyse at a high rate CD56+ SCLC and other CD56+ malignancy cell lines, with high specificity and achieving up to 64.9% of specific lysis. Moreover, they demonstrated that in xenograft mouse models infused with SCLC cell lines, CD56 expression facilitated tumor-cell killing, since mice bearing CD56+ tumors experienced a considerable reduction in tumor burden after 20 days after tumor-cell injection and an increased OS, suggesting a potential immunotherapeutic approach for CD56+ SCLC. We retrieved a single registered study (NCT03392064) on the use of CAR-T in SCLC. This is a phase 1 study aimed to evaluate the safety and tolerability of AMG 119, a CAR-T targeting delta-like protein 3 (DLL3), in SSLC patients who radiographically documented disease progression or recurrence after at least one platinum-based chemotherapy regimen. DDL3, an inhibitory Notch ligand, has been demonstrated to be highly expressed in SCLC, and it has therefore been explored as a potential therapeutic target for SCLC patients [45,46]. The primary outcomes include the incidence of dose-limiting toxicities, the incidence of treatment-emergent adverse events, and treatment-related adverse events. Among the secondary outcome measures, objective response, DOR, PFS, 1-year OS, and OS have been reported. The study started on 10 September 2018, with an estimated enrolment of 6 participants, and the estimated study completion date is 13 January 2026. Preliminary data on this study (NCT03392064) has been recently presented at the 2022 Annual Meeting of the American Society for Clinical Pharmacology and Therapeutics by Zhou and colleagues [47]. Five SCLC patients were included and all of them received AMG 119 therapy at two different doses (cohort 1: 3 × 105 CAR-T cells/kg, 3 patients; cohort 2: 1 × 106 CAR-T cells/kg, 2 patients). In both cohorts, AMG 119 determined a significant cellular expansion with long-lasting cell persistence (up to 86 days) and resulted in a well-tolerated treatment.

### 2.3. Neuroendocrine Prostate Carcinoma

NEPC is an aggressive variant of prostate cancer that may arise de novo or as a tumor evolution following hormonal therapies for prostate adenocarcinoma [48]. The incidence of neuroendocrine phenotypes in primary prostate cancers is approximately 1%, whereas, in lethal metastatic castrate-resistant prostate cancers, its percentage reaches 30% [49]. The prognosis of NEPC patients is poor, representing this neoplasia as the most lethal prostate cancer [50], with a median cancer-specific survival of 7 months [51]. According to NCCN guidelines, first-line therapy of NEPC consists of platinum-based combinations with taxanes or etoposide [52]. The combination of carboplatin with cabazitaxel is particularly useful for patients characterized by unfavorable genomics (i.e., loss of function mutations in PTEN, TP53, and RB1 genes); at the same time, the carboplatin–etoposide regimen is preferred for patients with pure small cell carcinomas [49]. Based on clinical and pathological features, second-line or alternative treatments could also be proposed as valuable and effective therapeutic options [53]. Moreover, in this cancer, CAR-T cell-based immunotherapy might be a promising strategy, and efforts have been made to identify NEPC-molecular targets [54,55]. The pioneering work of Lee et al. clearly demonstrated that divergent cancer differentiation states arising during prostate cancer progression are associated with changes in the expression of cell surface proteins [56]. Performing high-throughput multi-omic analyses, generated combined integrated transcriptomic and cell-surface proteomic data, and they identified carcinoembryonic antigen-related cell adhesion molecule 5 (CEACAM5) as an ideal NEPC target antigen. To test its therapeutic potential, they engineered CARs targeting CEACAM5 using lentiviral vectors and tested the efficacy and safety of these constructs in adenocarcinoma and NEPC cell lines and in patient-derived xenografts models. To quantify cytotoxicity, a co-culture assay using two engineered NEPC cell lines (MSKCC EF1—CEACAM5− or NCI-H660—CEACAM5+) transduced with CEACAM5 CAR was employed. Co-culture of CEACAM5 CAR-transduced T cells with NCI-H660 led to >80–90% cell death by 48 h, while co-culture with the MSKCC EF1 caused a minor reduction in cell viability probably due to low levels of CEACAM5 expression in the MSKCC EF1 NEPC cell line. These preliminary encouraging data led Baek and co-workers to develop a monoclonal antibody named 1G9, targeting the membrane-proximal region of CEACAM5 [57]. To assess in vitro CAR-dependent cellular cytotoxicity, anti-CEACAM5 CAR-T cells were co-cultured for 24 h with CEACAM5 + NCI–H660 and Du145-CEACAM5-NEPC cell lines. Anti-CEACAM5 CAR-T displayed high cytotoxicity in CEACAM5 + NEPC cells, and the cell killing was attributed to an increased release of IL-4, GM-CSF, and GrzB/perforin. They further evaluated the in vivo cytotoxicity in mouse xenograft models of Du145 and Du145-CAECAM5 cells. hIgG1-1G9 treatment significantly slowed tumor growth and improved mouse survival compared to control mice. Together, their results show that the newly developed anti-CEACAM5 CAR-T was able to induce in vitro and in vivo suppression of NEPC growth. Even though the results coming from this research suggest a potent antitumor effect of CEACAM5 CAR-T, studies in humans are not presently ongoing.

### 2.4. Pancreatic Neuroendocrine Neoplasms

In order to overcome the scarcity of available tumor-associated antigens (TAAs) that are a known target for CAR-T cell therapy in NENs, in a recent study, Feng and colleagues [58] developed an unbiased method to identify potential new TAAs that could be targeted by CAR-T cells. The authors used a phage display screening method to identify camelid animal-derived single variable domain antibodies (VHH or nanobodies) that preferentially bind to the surface of gastrointestinal (GI)-NET cells. As a result, they isolated the nanobody VHH1, which selectively binds to BON1 pancreatic neuroendocrine cells. VHH1 showed to specifically target CDH17, a cell surface adhesion protein with known overexpression in GI-NETs [59,60]. The authors demonstrated, by in vitro and in vivo experiments (using autochthonous mouse models), that VHH1-CAR-T cells (CDH17 CAR-Ts) were cytotoxic to both human and mouse tumor cells in a CDH17-dependent manner. The authors compared three CDH17 CAR-Ts approaches in three mice cohorts who were engrafted with NT-3, a pancreatic islet CDH17-expressing NET cell line, developing CDH17 + NT-3 tumors. Subsequently, after 35 days from the xenograft, CAR-T cells were infused, and the treatment was repeated after a further 5 days. The first group of mice was subjected to infusion with CDH17 CAR-Ts containing CD28 and 4-1BB costimulatory domains (VHH1-28BBz), the second group with CDH17 CAR-Ts without CD28 and 4-1BB costimulatory domains (VHH1-BBz) (Figure 1) and the third group with untransduced T Cells (CDH17-UTD). The growth curve of the tumors in each group of the mice was assessed: VHH1-28BBz CDH17 CAR-T cells induced tumor mass reduction until complete eradication of all tumors after 42 days; VHH1-BBZ showed progressive volume reduction but were not able to cause tumor elimination; conversely, CDH17-UTD therapy failed to control tumor growth. The tumors were also dissected and analyzed 10 days after the first infusion. In particular, tumoral tissues treated with VHH1-28BBz CDH17 CAR-Ts revealed the absence of neuroendocrine cells while abundant T-Cells were detected, demonstrating rapid tumor elimination.

Based on the wide and peculiar overexpression of SSTRs on neuroendocrine cancer cells [61], as well as the established efficacy in clinical practice of SSA and radiolabeled SSA in advanced NENs treatment, Mandriani and colleagues [62] developed CAR-T cells to directly target SSTRs. The innovative CAR-T construct included two molecules of the SSA octreotide, which binds with high affinity to SSTR2 and SSTR5, and the costimulatory molecule CD28. The CAR-Ts were cloned in a retroviral vector and subsequently transduced in CD8+ cells. CAR-T cells were then co-incubated with different human NEN cell lines from pNEN, namely BON1, QGP1, and CM, that were previously screened for SSTR 1–5 overexpression. In vitro cytotoxicity was assessed by bioluminescence after 72 h of co-culture, showing tumor cell death in 58% (±8%), 53% (±1%), and 42% (±3%). In the same study, the authors evaluated the anti-SSRT CAR-T therapy effects in mice, subcutaneously engrafted with two different SSTR + NET cell lines (BON1 and CM). When NET xenografts reached 1 mm 3, mice were randomized to receive phosphate-buffered saline (PBS), UTD T cells, or anti-SSTR CAR-T cells by tail vein injection. In vivo tumor growth was assessed by bioluminescence. Mice treated with anti-SSTR CAR-T cells showed a significant reduction in tumor growth as compared with the animals treated with UTD T cells or PBS; the difference in tumor growth reached statistical significance (*p* < 0.05) after 14 and 21 days for CM and BON1 tumors, respectively. No evident adverse effects to the animals were detected up to 4 weeks after treatment

### 2.5. Ileal and Lung Neuroendocrine Cells

Finally, the study above reported (61) that CAR-T cells were co-incubated with human NEN cell lines from intestinal NET (CNDT2.5) and lung carcinoid (H727). In vitro cytotoxicity showed tumor cell death in 37% (±7%) and 31% (±14%), respectively. No in vivo data are reported.

**Figure 2:**
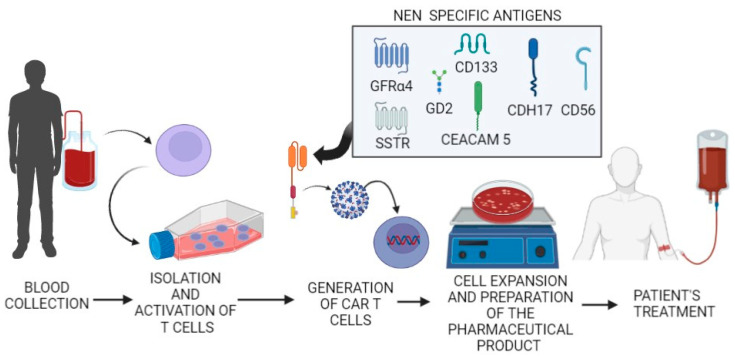
The process of developing CAR-T cells as a therapeutic approach for NEN.

Figure 2 summarizes the CAR-T cells against NEN antigens (GFRα4, CD133, GD2, CD56, CEACAM5, CDH17, and SSTR) developed in the last few years.

T cells are isolated from a patient’s blood (or from a donor), are expanded, and then activated in vitro. The culture is then genetically engineered to express the CAR constructs (in the square are reported target antigens used in the preparations directed against NEN, as discussed in the text). CAR-T cells undergo to further in vitro expansion and are prepared as a pharmacological product that will be administered to the patient.

### 2.6. Potential Future Applications

Potentially interesting data may also arise from studies not specifically designed for NENs. For example, 3 phase I and Ib clinical trials developed by Katz and colleagues [63,64,65] evaluated the use of regional administration of anti-CEA CAR-T cell therapy targeting CEA^+^ liver metastasis from digestive tract adenocarcinomas through intra-hepatic artery infusion, showing clinical efficacy and safety.

Interestingly, CEA is known to be overexpressed in MTC and other different types of NENs [66,67]. Clinically, excellent targeting of MTC has been found with radiolabeled anti-CEA antibodies, and antitumor effects have been achieved with ^131^I-labelled anti-CEA antibodies, suggesting a high potential of pretargeting for diagnostic and theranostic applications in MTC patients [68,69,70,71,72]. Thus, anti-CEA CAR-T cell therapy might be potentially useful in refractory MTC and/or other CEA^+^ NENs.

## 3. Conclusions

In the last few years, preclinical studies performed on NEN cell lines and NEN animal models suggested the potential relevance of CAR-T cells for NEN treatment. Ongoing and future clinical trials will clarify their actual efficacy in these tumors. Given the limitations of safety [73,74] and costs (possibly reaching and/or exceeding $400,000) [75], the assessment of the health outcomes versus alternative therapies will represent a crucial point. The chance of such an individualized approach should then be preferred, rather than as a general approach, only in selected patients with highly aggressive NENs, lacking other therapeutic options, as already occurs in hematological malignancies and other solid tumors.

## Figures and Tables

**Table 1:** Current FDA/EMA approved CAR-T cells.

Brand Name	Generic Name	Target	Indications	FDA Approval (Year)	EMA Approval (Year)
Abecma	idecabtagene vicleucel	B-cell maturation antigen	relapsed/refractory multiple myeloma	2021	2021 (conditional)
Breyanzi	lisocabtagene maraleucel	CD19	relapsed/refractory diffuse large B-cell lymphoma, primary mediastinal large B-cell lymphoma, follicular lymphoma grade 3B, after two or more lines of systemic therapy	2021	2022 (initial orphan drug approval 2017)
Carvykti	ciltacabtagene autoleucel	CD38	Relapsed/refractory multiple myeloma	2022	2022 (orphan)
Kymriah	tisagenlecleucel	CD19	B-cell acute lymphoblastic leukemia, relapsed or refractory diffuse large B-cell lymphoma and follicular lymphoma	2017	2018 (initial orphan drug approval 2016)
Tecartus	brexucabtagene autoleucel	CD19	relapsed/refractory mantle cell lymphoma	2020	2019 (conditional)
Yescarta	axicabtagene ciloleucel	CD19	diffuse large B-cell lymphoma, transformed follicular lymphoma, primary mediastinal B-cell lymphoma	2017	2018 (initial orphan drug approval 2016)
